# Effect of Compatibility on the Foaming Behavior of Injection Molded Polypropylene and Polycarbonate Blend Parts

**DOI:** 10.3390/polym11020300

**Published:** 2019-02-11

**Authors:** Bei Su, Ying-Guo Zhou, Bin-Bin Dong, Cao Yan

**Affiliations:** 1 School of Materials Science and Engineering, Jiangsu University of Science and Technology, Zhenjiang, Jiangsu 212003, China; subei_2005@126.com (B.S.); yanchao@just.edu.cn (C.Y.); 2 National Engineering Research Center for Advanced Polymer Processing Technology, Zhengzhou University, Zhengzhou, Henan 450001, China

**Keywords:** compatibility, PP/PC, foaming, injection molding

## Abstract

To improve the foaming behavior of a common linear polypropylene (PP) resin, polycarbonate (PC) was blended with PP, and three different grafted polymers were used as the compatibilizers. The solid and foamed samples of the PP/PC 3:1 blend with different compatibilizers were first fabricated by melt extrusion followed by injection molding (IM) with and without a blowing agent. The mechanical properties, thermal features, morphological structure, and relative rheological characterizations of these samples were studied using a tensile test, dynamic mechanical analyzer (DMA), scanning electron microscope (SEM), and torque rheometer. It can be found from the experimental results that the influence of the compatibility between the PP and PC phases on the foaming behavior of PP/PC blends is substantial. The results suggest that PC coupling with an appropriate compatibilizer is a potential method to improve the foamability of PP resin. The comprehensive effect of PC and a suitable compatibilizer on the foamability of PP can be attributed to two possible mechanisms, i.e., the partial compatibility between phases that facilitates cell nucleation and the improved gas-melt viscosity that helps to form a fine foaming structure.

## 1. Introduction

Polypropylene (PP) foams are often viewed as one of the most popular lightweight thermoplastic materials. This is partially attributed to their advantages, such as low density, low material cost, high heat distortion temperature, excellent chemical resistance, and potential degradation ability. These properties provide support for industrial and agricultural applications, including parts for the automotive industry, packaging, appliances, and electronics [1,2]. However, pure PP is often reported to be unsuitable for foaming processing due to its linear structure and low melt strength, leading to cell rupture/coalescence [3,4]. Specifically, it is also realized that it is relatively difficult to fabricate a foamed sample of pure PP resin with a fine microcellular structure using the injection molding (IM) method compared to the ideal conditions in batch foaming processing [5]. This may be attributed to the difficulty of obtaining the correct balance of appropriate IM parameters and suitable foaming conditions. For example, it has been reported that the appropriate foaming temperature range of linear PP resin is only approximately 4 °C [6]. However, it is also well known that a large variation of temperature is often needed for successful IM operation. Broadening the foaming window can be a popular method for improving the flexibility of pure PP foaming, and involves self-modifying its melt strength via ionic modification [7] and crosslinking [8], adding micro- or nanoparticles or fibers into the PP matrices as nucleating agents [9,10,11,12,13], and utilizing other polymers as fillers [14,15,16,17,18,19,20,21,22,23,24,25]. However, it has been shown that self-modification generally involves a complex chemical synthesis, and micro- or nanoparticles are also difficult to disperse uniformly. Hence, compared to these two methods, blending with other polymers could possibly be a relatively economical and effective method to enhance the foamability of pure PP resin. For example, it has been reported that PP foaming ability could be significantly enhanced by blending with polystyrene (PS) [14,15], high-density polyethylene (HDPE) [16,17,18], low-density polyethylene (LDPE) [19,20,21,22,23], ethylene-octene copolymer (EOC) [24], and acrylonitrile-butadiene-styrene (ABS) terpolymer [25]. The multiphases of these blend systems could decrease the gas bubble nucleation energy, which thus improves the cellular foaming structures. As a result, foaming of two- or multi-phase blends can be identified as a promising approach to satisfy the steadily growing demand for PP foams with enhanced properties. 

Polycarbonate (PC) is an important engineering thermoplastic polymer. It possesses exceptional properties, such as flame resistance, toughness, ductility, impact strength, and a very wide serviceable temperature range. Blending PP with PC, if it results in an improved foamability and mechanical properties of the PP matrix, may become an economical and effective method to produce new foamed parts for specific applications. In the past decade, PP and PC have been blended together and applied because of the combined advantages of the two resins [26,27]. However, PP and PC are very immiscible and are incompatible with each other, resulting in poor mechanical properties for PP/PC blends. For example, the poor fracture toughness of the PP/PC blends is unacceptably lower than for the PP and PC resins [28]. 

To overcome the limitation of PP/PC blends due to the poor compatibility between the PP and PC phases, effective compatibilizing was evaluated because it is a key to obtaining materials with elevated mechanical properties [29,30,31]. Adding compatibilizers is often viewed as a conventional method to improve the poor interfacial bond strength between two or multiple phases of polymer blends [32]. It has been proven that the incorporation of a suitable compatibilizer into the PP/PC blends can improve the mechanical properties of PP/PC blends involved in most polymer processes, such as IM [33], extrusion [34], 3D printing [35], and even annealing [36]. If the compatibility of the PP/PC phases is adjusted appropriately, it is expected that the blends could be used to produce foamed injection molded parts. Furthermore, from the perspective of promoting foaming, the incompatibility between PP and PC seems to provide more opportunities for nucleation and actually facilitate PP/PC foams. It is also possible that the difference of foaming behavior between PP and PC resin substantially influences the cellular structure of PP/PC blends. In addition, compared to the reported blended polymers such as PS, PE, and ABS mentioned before, PC has a higher viscous flow temperature except for its more excellent mechanical properties, which may result in an unexpected effect on the foaming behavior of PP resin. Therefore, it is expected that the addition of PC can modify and even improve the foaming behavior of pure PP resin. In our previous work [37], a method to fabricate PP/PC microcellular parts was proposed, and the effect of the addition of PC on the foaming behavior of PP resin can be hypothesized. Such a case aroused our curiosity to thoroughly explore the foaming behavior of PP/PC blends in IM processes. 

However, compared to conventional IM, the IM operations with a physical or chemical blowing agent are more complex, which involves generally five steps of the foaming process, i.e., gas generation, gas dissolution, cell nucleation, cell growth, and cell shaping. All the five steps must occur in a popular-used IM machine with a proper mold, and the total foaming process rely on the right processing technologies once the equipment and material are selected. Although technical details of IM for microcellular and solid parts have been investigated for decades [38,39,40,41,42,43,44,45,46,47,48,49], the same issues have scarcely been investigated for the foaming behavior of PP/PC blends using the real IM operations yet. 

Therefore, combining the sophisticated fields of PP/PC blends and foaming IM processes not only offers great opportunities but also poses significant challenges, as the multiphase characteristics of PP/PC blends and the complexity of foam processing need to be carefully considered. It is necessary to adjust and control the compatibility of the PP/PC phases to produce parts made of PP/PC foam with improved cellular structures and suitable mechanical properties. In this study, a method to improve the foamability of PP resin via blending with PC was introduced, and the effect of compatibility of the two phases on the cellular structure of PP/PC foams was investigated. After comparing the experimental results of the neat resins and the different PP/PC blends, a better understanding of the influence of the compatibility on the cellular morphology of PP/PC blends was gained. 

## 2. Experimental

### 2.1. Materials

**Figure 1:**
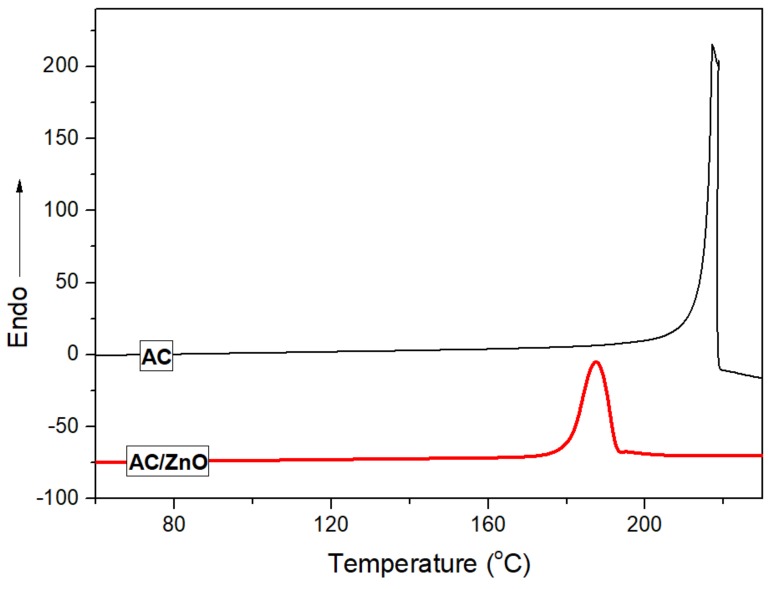
The heating flow curve of AC and AC/ZnO at a heating rate of 10 °C/min.

The PC (Lexan 141R, SABIC, Riyadh, Saudi Arabia) and PP (Huajin, Panjing, China, T30S) used in this study were commercial products. Their melt volume flow rates were about 12.0 cm^3^/10 min (300 °C, 11.8 N) and 3.2 cm^3^/10 min (190 °C, 21.6 N), respectively. For brevity, the ratio of PC and PP in PP/PC blend was fixed at 3:1 by weight. Polypropylene-graft-maleic anhydride (PP-*g*-MA, with an MA of 8.0 wt %), poly(propylene-*co*-glycidyl methacrylate) (PP-*c*-GMA, with a GMA of 8.0 wt %), and ethylene-acrylic ester-glycidyl methacrylate (E-MA-GMA, with a GMA of 8.0 wt %) were used for the compatibilizer separately. Their melt volume flow rates were approximately 5 cm^3^/10 min, 5 cm^3^/10 min, and 6 cm^3^/10 min (190 °C, 21.6 N), respectively. The above materials were dried in an oven at 60 °C for 10 hours to remove any possible moisture before use. In addition, the azodicarbonamide (AC, Dn8) was used as a blowing agent, and zinc oxide (ZnO) was used as a blowing auxiliary agent. To seek a decomposition temperature that is suitable for PP/PC blends, the ratio of AC and ZnO was fixed at 1:0.0068 after many experiments. The thermal decomposition behavior of the original AC powder and AC/ZnO composite was characterized using a differential scanning calorimetry (DSC) test with a scan rate of 10 °C/min, as shown in Figure 1. 

It can be seen from Figure 1 that the decomposition temperature of the original AC powder was in the range 194~220 °C with a peak maximum at approximately 205 °C, and the decomposition temperature of the AC/ZnO composite decreased to 175–193 °C with a peak maximum of approximately 188 °C, which was proven to be more suitable for the foaming IM of PP/PC blends used in this study. The gases released during the decomposition of the AC/ZnO composite were N_2_ (65%), CO (25%), CO_2_ (5%), and NH_3_ (5%). For convenience, a foaming masterbatch including the AC/ZnO composite coupled with a dispersing agent was fabricated using the PP resin for the matrices. 

### 2.2. Sample Preparation

The sample preparation included two steps: PP/PC blend granulation and sample fabrication. During blend granulation, the dried PP and PC were first blended using a high-speed mixer under room conditions for approximately 2 minutes and then extruded using a twin-screw extruder with an appropriate draw ratio. The main processing parameters of the mixing and the extrusion are listed in Table 1. The extruded materials were pelletized and dried for the subsequent manufacturing. In this stage, the compositions of the PP/PC blends by weight percentage were fixed at 75:25 for brevity. PP-*g*-MA, PP-*c*-GMA, and E-MA-GAM were added and then mixed with PP/PC blend. After a series of experiments and comparisons, their weight content in the PP/PC blend was fixed at 10%. As a result, four types of PP/PC blends were obtained, i.e., PP/PC 3:1, PP/PC/PP-*g*-MA (30:10):4, PP/PC/PP-*c*-GMA (30:10):4, and PP/PC/E-MA-GMA (30:10):4. These blends were named PPC, PCM, PCG, and PCE in this study, respectively. The pure PP and PC resin were used for comparison with the same thermal mechanical history conditions, and consequently, six PP/PC pellets were obtained. 

The foamed samples were then fabricated using the six PP/PC blends in a commonly used IM machine with the addition of the foaming masterbatch. It must be noted that, as mentioned before, it is not easy to fabricate a foamed sample with fine cellular structure using the IM method with the AC foaming masterbatch. Compared to conventional IM, the foaming IM involves more processing parameters such as the dosage of the foaming masterbatch and the shot size. These can deeply influence part quality. The active ingredient of AC in PP/PC blends was determined to be 1.05% and a range 90%~93% of full shot size was used in this study, and they hence produce a weight reduction of appropriately 7%~10%. Furthermore, after mixing the foaming agent, the neat polymer melt in conventional IM changes into a gas-melt solution in foaming IM. The control of a two-phase solution is more complex than a single-phase melt using the same common IM machine. Therefore, it is of critical importance to effectively control the processing parameters during the IM procedure for the best quality of a microcellular part. 

**Figure 2:**
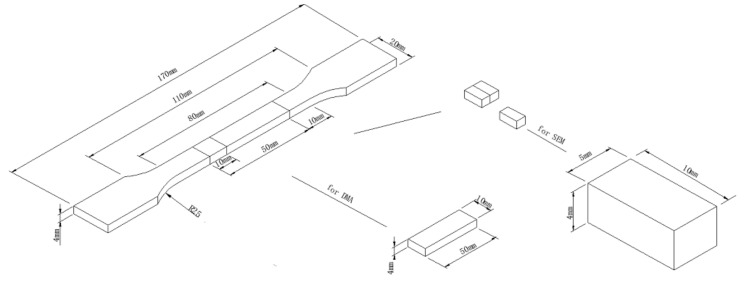
A detailed description of sample size and preparation.

It is well known that the processing parameters during IM mainly include the melt temperature, mold temperature, injection pressure, injection rate (time), packing pressure, packing time, and cycle time. For the foaming IM used in this study, an appropriate range of temperatures and times needs to be set because the foaming masterbatch will decompose very rapidly once the temperature increases, and decomposition will slow or even stop when the temperature decreases. After many tests, the melt temperature and cycle time were determined as 230 °C and 40 s, respectively, which were found to be suitable for obtaining the correct balance of the decomposition of the foaming masterbatch and the IM operation. Furthermore, the injection volume rate is also an important parameter of the IM for the quality of the foaming process. It is generally believed that the pressure drop rate increases significantly with the rise of the injection volume rate, which results in an increase of the nucleation rate [50]. Therefore, both high injection rate and large injection pressure were selected in this study to ensure the rapid injection process. In addition, the packing pressure and/or time are often viewed as some of the most important factors for the conventional IM process. They are equally important for the foaming IM. Different packing pressures and/or times can produce different foamed structures inside of the part which will lead to different microstructures and hence different mechanical properties. An appropriate packing pressure and time were found to be facilitate the foaming IM process [21], and they were determined as 10 MPa and 1 s in this study, respectively. As to the conventional IM process, a high packing pressure and time is often necessary to avoid the shrinkage of the molded parts. The selective processing parameters are listed in Table 2. For comparison, solid samples were prepared with the same thermal-mechanical history of the foamed samples but without the foaming masterbatch, and the packing pressure was fixed at 75 MPa and the packing time was 5 s. A standard tensile test bar mold was used to mold the samples, and its temperature was controlled by circulating oil from a thermal controller. The volume of the cavity of the mold was about 9.58 × 10^3^ mm^3^ and a detailed description of the molded sample is shown in Figure 2. 

### 2.3. Sample Tests

Tensile tests were carried out according to ASTM D638-10 standards using a screw-driven universal testing instrument (MTS, Eden Prairie, MN, USA, Sintech 10/GL) under ambient conditions. Crosshead speeds of 10 mm/min were used to study the stress and strain behavior of the molded tensile samples. Seven tensile bars were tested for each material, and the biggest and the smallest values were excluded. Hence, there were five values selected for analyzing, and the mean and range of ultimate tensile strength and strain-at-break for each group of samples were calculated and reported. 

The morphologies of the selected molded specimens were examined using a scanning electron microscope (SEM JEOL JSM-6480, Tokyo, Japan) with an accelerating voltage of 20 kV. The SEM specimens, which are also shown in Figure 2, were taken from the cross-section of the molded tensile bar that was fractured in liquid nitrogen. The surfaces of the fractured specimens were sputter coated with gold prior to observation for a period of 60 second. The coating equipment used is an auto sputter coater (Cressington 108, Watford, UK). 

Furthermore, the quantitative analysis based on the information of the cell morphology provided by the SEM pictures were performed using an image processing tool of the ImageJ® software package (Bethesda, MA, USA). The cell diameter could be calculated with the hypothesis of spherical shape cells after the area of each cells in the SEM picture was assessed, and then the mean cell size of the foams could be evaluated. Cell density—the number of cells created per unit volume (cm^3^)—in the foamed samples could be calculated by using the following equation [51]:

(1) $$N={\left(\frac{n}{A}\right)}^{3/2}\times \frac{\rho_{s}}{\rho_{f}}$$

where *n* is the number of cells in the SEM micrograph, *A* is the area of the micrograph (cm^2^), and *ρ*_s_ and *ρ*_f_ are the density of the solid and foamed materials (g/cm^3^). The density of the solid and the foamed samples was determined by water displacement method according to ISO 1183-1987, respectively, and the results of the six kinds of PP/PC blends are listed in Table 3. A 10%–12% decrease of the density can be found by the comparison between the solid and the foamed samples. 

To determine the interaction on a molecular level between the different components in the polymer blend, dynamic mechanical properties were studied using a dynamic mechanical analyzer (DMA 242E NETZSXCH, Selb, Germany) instrument. As shown in Figure 2, the gauge length of the standard tensile test sample was 80 mm. Hence, rectangular bars with a 50 mm length, 10 mm width, and 4 mm thickness could be obtained for the DMA test by slicing the gauge section of the tensile bar. 

The rheological tests were carried out on a torque rheometer (Haake System 90, Thermo Fisher Scientific, Karlsruhe, Germany) with a measure head (mixing room) of 60 cm^3^. The rotation speed relation between rollers was 2/3. The different composition of PP/PC blends with and without the addition of the foaming masterbatch were tested at 230 °C under air atmosphere. The measuring head was generally loaded to 90% of its volume capacity during the entire test to avoid the influence of apparent filling degree on torque [52,53].

## 3. Results and Discussion

### 3.1. Mechanical Properties

**Figure 3:**
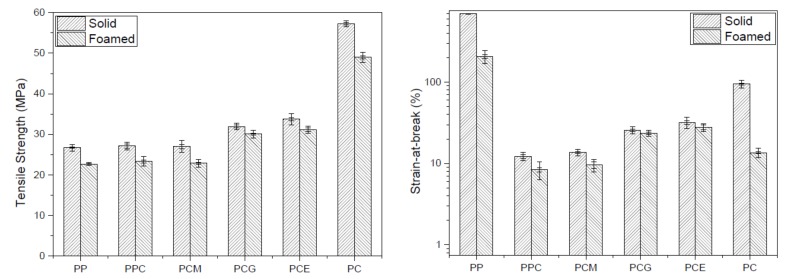
The tensile strength (**a**) and strain-at-break (**b**) of the molded solid and foamed PP/PC samples.

Tensile tests were performed for the PP/PC blend solid and foamed samples, and the results of the tensile strength and the strain-at-break are shown in Figure 3. Figure 3 shows that the tensile strength of the mold solid PPC samples is almost equal to that of the PP samples. The strain-at-break even shows a drastic reduction with the addition of the PC. It suggests that the incorporation of a more rigid PC phase into the PP matrix significantly reduces the material’s ability to be extended [28]. Hence, it can be concluded that the addition of PC did not improve the mechanical properties of the PP material effectively. This is attributed to the weak interfacial interaction between the PP and PC phases, which cannot prevent the initiation and propagation of cracks along the interface between the two phases. Furthermore, it is well known that the tensile strength of compatibilizers themselves is generally low. However, with the addition of different compatibilizers, the mechanical properties show different trends. It can be seen from Figure 3 that the addition of 10.0 wt % PP-*c*-GMA to the PP/PC blend solid samples causes a substantial improvement in the tensile strength and the strain-at-break of approximately 17.8% and 110.4%, respectively. For PCE, the increases in the tensile strength and the strain-at-break are as high as 24.4% and 162.4%, respectively. Compared to these two groups of blends, the addition of PP-*g*-MA causes a slight decrease in the tensile strength of 4.8% and an increase in the strain-at-break of 12.0% of PPC solid samples. The poor mechanical properties of the PCM are understandable because of the poor compatibilizing effect of PP-*g*-MA on PP/PC blends. Furthermore, the improved tensile strength and strain-at-break of the PCG and PCE samples can be attributed to the increased compatibility of the PP and PC phases. The effect of the compatibilizers on the mechanical properties of PP/PC blend foamed samples can also be found in Figure 3, and the influence on the foaming and solid samples was similar. 

**Figure 4:**
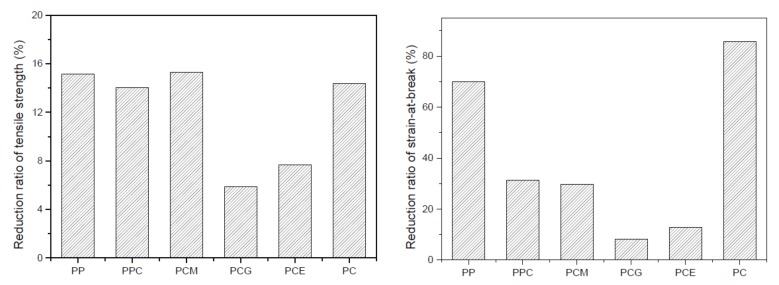
Reduction ratio of tensile strength (**a**) and strain-at-break (**b**) of the molded foamed PP/PC samples compared to the solid ones.

It can also be observed from Figure 3 that the mechanical properties of the foamed samples of PP/PC blends, regardless of whether compatibilizer was added into or not, are generally lower than that of the solid samples. Generally, the original cross-sectional area of foamed samples is smaller than that of solid ones owing to the existence of foamed pores. However, the reduction of cross-sectional area was ignored in the calculation of the nominal tensile strength used in this study. Therefore, the decrease of tensile strengths is expected, since the nominal tensile strength was obtained directly by dividing the maximum load by the original cross-sectional area. However, it can be found that there is a different reduction ratio of the foamed sample to the solid one for the six types of different PP/PC blends. To make it clearer, the reduction ratio was proposed, which can be calculated using the following expression:

(2) $$R_{m}=\frac{M_{s}-M_{F}}{M_{s}}\times 100\%$$

where *R*_m_ is the reduction ratio of the mechanical property (*M*) of foam samples relative to solid samples, and *S* and *F* are the solid sample and foamed sample, respectively. The reduction ratio of the tensile strength and the strain-at-break of the six PP/PC blends are plotted in Figure 4a,b, respectively. An interesting phenomenon can be observed from Figure 4, whereby the addition of PP-*c*-GMA or E-MA-GMA causes a clear decrease in the reduction ratio of the tensile strength and the strain-at-break, while the addition of PP-*g*-MA has almost no influence. It is believed that the morphological cell structures can mostly explain the reasons for the variations in the mechanical properties for different foamed samples. Hence, the microstructures of the microcellular parts are further investigated in the following section. 

### 3.2. Foaming Behavior

**Figure 5:**
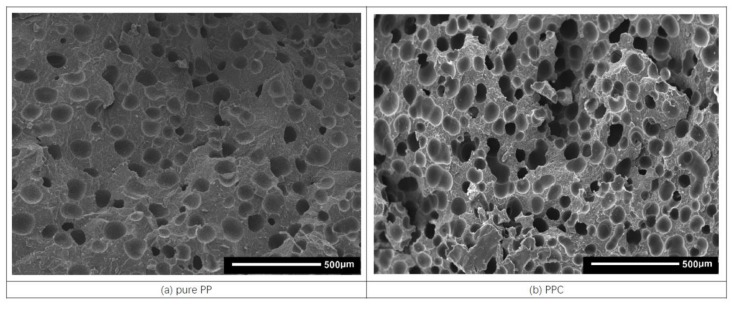

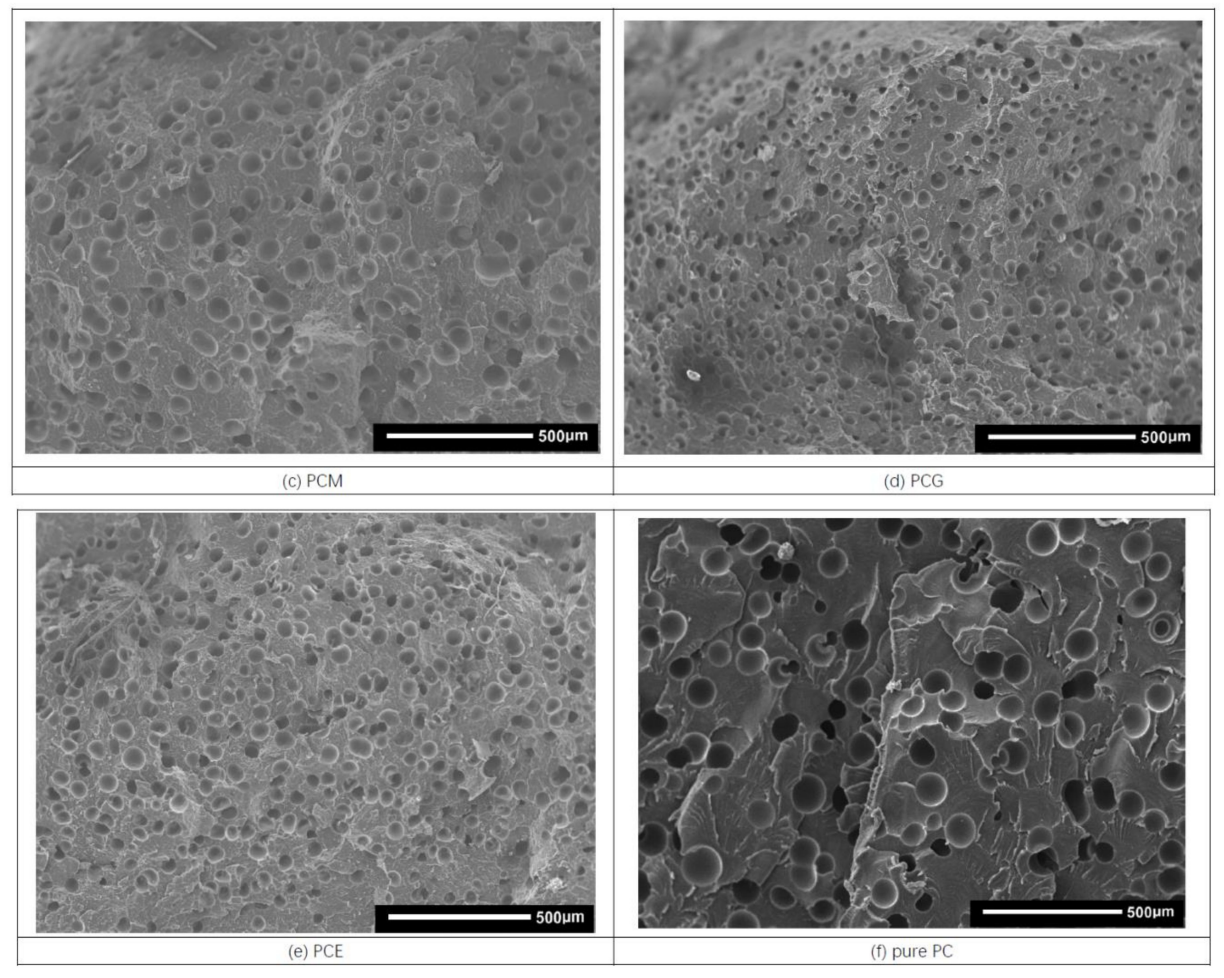
SEM images of the six selective foamed PP/PC samples. (**a**: pure PP; **b**: PPC; **c**: PCM; **d**: PCG; **e**: PCE; **f**: pure PC).

It is well known that SEM observation is one of the most popular and reliable ways to check morphological characteristics, which are generally viewed as the determining factors of the final physical and mechanical properties of microcellular foamed parts and include the cell density, size, and distribution. Figure 5 shows the SEM images of the fractured cross sections of the six representative foamed PP/PC blend samples. It can be observed from Figure 5 that the detailed cellular structures are clearly different owing to the addition of PC and compatibilizers. Compared to the neat PP resin shown in Figure 5a, the number of cells substantially increase with the addition of PC, as shown in Figure 5b, which means that the addition of PC could improve the foaming cell density of pure PP resins. Furthermore, the influence of different compatibilizers on the cellular morphology of the PP/PC blend can be found from the comparisons among Figure 5c–e. The addition of PP-g-MA has almost no effect on the foaming behavior of the PP/PC blends. Comparatively, the cell density increases and the cell size decreases when PP-*c*-GMA (or E-MA-GMA) is added into the PP/PC blends, as shown in Figure 5d (or Figure 5e), indicating a uniformly distributed foamed structure formed. Hence, the combined effect of PC and PP-*c*-GMA (or PC and E-MA-GMA) could facilitate the foaming behavior of PP resins. After compared to PCE and other PP/PC blends, the foamed structure of PCG shown in Figure 5d is probably the most uniform among all six images shown in Figure 5. It can also be seen from Figure 5 that all foams exhibit a completely closed cell cellular structure, which can ensure that the mechanical properties of the samples do not drop too much as a result of the bubbles. 

**Figure 6:**
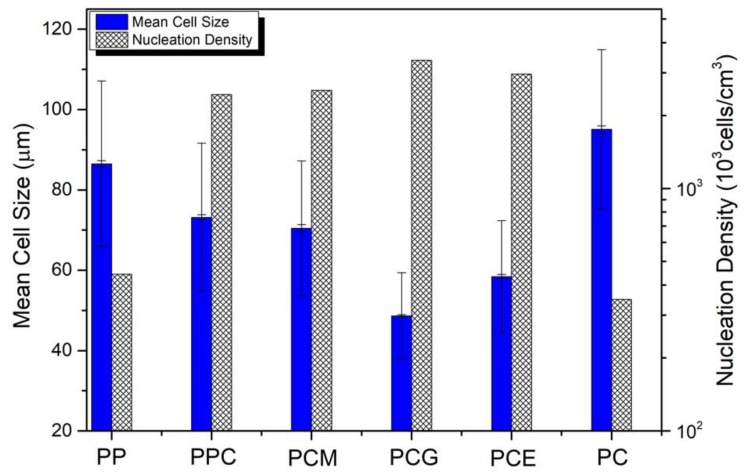
Statistic results of cell size and cell density from the SEM images of Figure 5.

Therefore, based on the direct observation and comparison in Figure 5, the following three conclusions can be drawn: (1) the addition of PC resulted in good foamability of the PP resin when the PP/PC blends were used in the foaming IM as described in this study; (2) the effect of PP-*g*-MA in improving the foaming behavior of PP/PC blend was not significant; and (3) the addition of PP-*c*-GMA or E-MA-GMA could improve the cellular structure of PP/PC blend. The statistical results of the mean cell size and cell density of PP/PC blend foams are shown in Figure 6, which supports the qualitative conclusions. It can also be found from Figure 6 that the PCG sample has the most uniform cell size distribution within the SEM images, which corresponds to the minimum reduction ratio of mechanical properties shown in Figure 4. Hence, it can be concluded that a fine foaming structure can lead to excellent mechanical properties. Furthermore, as mentioned before, it is not easy to fabricate a foamed PP sample with a fine cell density using the conventional IM method, in which the cell density is generally reported to be as low as 10^5^ cells/cm^3^ with a cell size larger than 100 µm for a neat PP resin [3,23,25]. In comparison, ideal batch foaming processing can result in a cell density as high as 10^8^ cells/cm^3^ [3]. In this study, the nucleation density is higher than 3.38 × 10^6^ (cells/cm^3^), and the cell size is as small as 48.6 µm, as shown in Figure 6. Therefore, the method of fabricating PP foamed components with the addition of PC and PP-*c*-GMA might be an effective way to improve the cellular structure of foamed IM parts that actually results in improved mechanical properties and saves materials. This approach can replace solid parts with a 10% material reduction without significantly compromising the required material properties. 

It can be concluded from the above discussion that the use of the different compatibilizers made a great difference in the morphological structure and mechanical properties, which can be attributed to the different effects from the compatibilizers and needs to be discussed further. 

### 3.3. Compatibility

**Figure 7:**
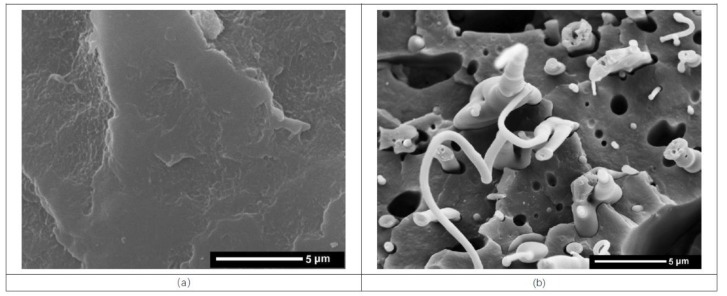

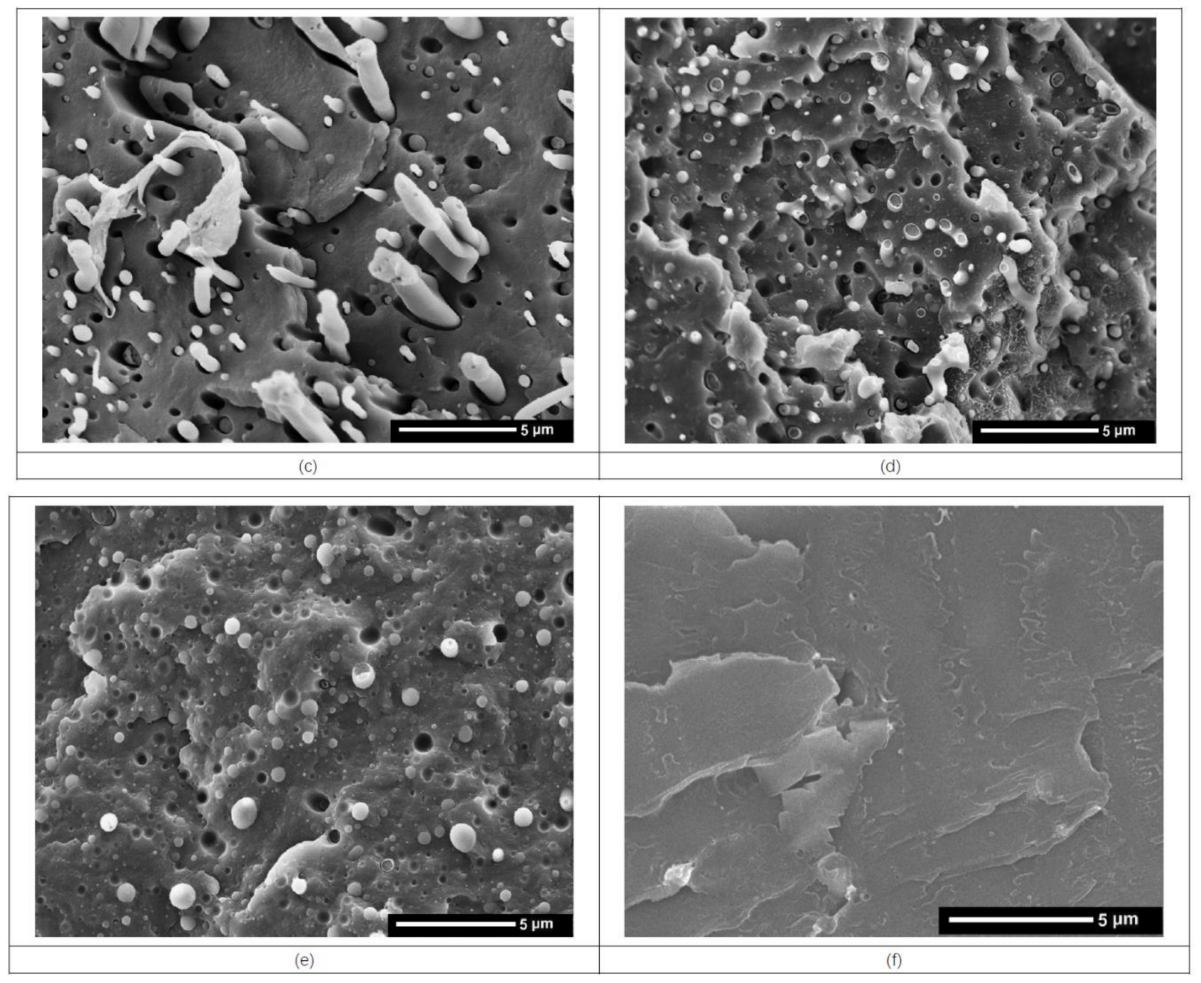
SEM images of the molded sample. (**a**: pure PP; **b**: PPC; **c**: PCM; **d**: PCG; **e**: PCE; **f**: pure PC).

The addition of a compatibilizer is a widely used and efficient method for enhancing the interfacial adhesion between phases of polymer blends, thus resulting in an improvement in the mechanical properties. SEM was used to characterize the PP/PC blend morphology, which is a well-accepted technique for examining the compatibility of polymer blends. Figure 7 shows SEM images of the fracture surfaces of the six representative samples. Figure 7a,f show the morphological characterization of the pure PP and PC resin, respectively, and both appear to be characteristic of a single homogeneous component. With the addition of PC, the two phases can be easily observed in Figure 7b, as PP clearly forms a continuous phase while PC forms large domains. It can also be observed in Figure 7b that most of the PC particles in the PP/PC blends are spherical. However, the size of the dispersed PC particles varies within a wide range. Not only that, there is an irregularly shaped dark region on the interface, which could be attributed to nonuniformly distributed PC droplets that were debonded and pulled out from the PP matrix during cryogenic deformation. Hence, it can be concluded from the heterogeneous and coarse phase dispersions at the PP/PC interface that the compatibility of PP and PC is not good enough to result in a low interfacial tension and stable adhesion between the PP and PC phases. Most likely, for this reason, the mechanical properties of the PP/PC sample are even worse than those of the pure PP resin, as discussed previously. 

When PP-*g*-MA was added to the PP/PC blend, as shown in Figure 7c, the poor distribution of the PC did not change significantly compared to the PPC sample. It can be concluded that PP-*g*-MA is a poor compatibilizer for PP and PC blends. Therefore, the addition of PP-*g*-MA cannot improve the mechanical properties of the PP/PC blend, which was observed previously. However, once PP-*c*-GMA was added, the effect is clearly different. Compared with the dispersion in Figure 7c, it can be shown in Figure 7d that the PC phase exhibits smaller particle diameters and a more regular and finer dispersion in the PP matrix, which indicates an increase in the compatibility between the PP and PC. As a result, the interfacial adhesion between the PP and PC phases improved in the presence of PP-*c*-GMA, subsequently reducing the interfacial tension between the two phases and leading to good mechanical properties. It was concluded that PP-*c*-GMA is an efficient alternative for improving the compatibility between PP and PC phases. 

However, the dispersion of the PC particles in the PP matrix does not appear to be thoroughly homogeneous, and the compatibility of the PP/PC is only partially improved with the addition of PP-*c*-GMA. The dispersion situation can be further improved with the addition of E-MA-GMA, which is shown in Figure 7e. Compared to the dispersion of the PC particles shown in Figure 7d, the PC particles shown in Figure 7e have a more uniform dispersion in the PP matrices, and the mean diameter of the dispersed PC particles also clearly decrease, which indicates a further increase in the compatibility between the PP and PC. For this reason, the tensile strength and the strain-at-break for the PCE solid samples have the highest value among the four compatibilized groups of PP and PC blends (including PPC, PCM, PCG, and PCE). 

**Figure 8:**
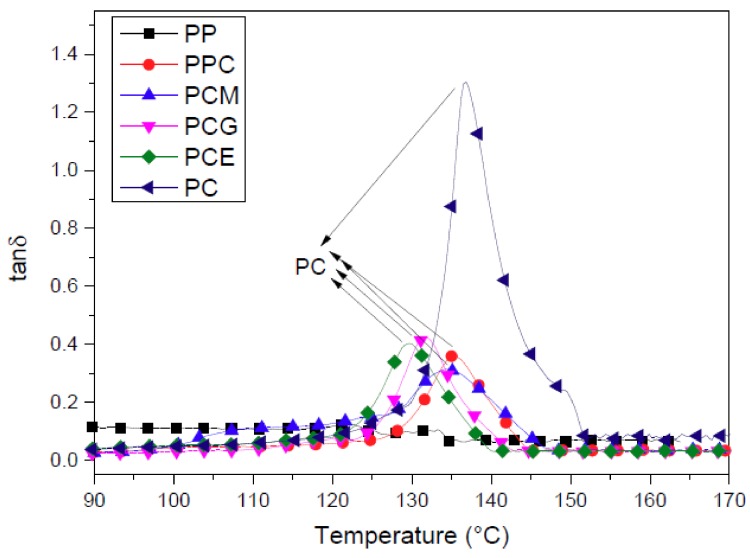
DMA curves (tanδ vs. temperature) of the six foamed PP/PC samples.

The compatibility was further examined by determining the glass transition temperature (*T*_g_) of the PC, which is shown as the loss factor vs. temperature curves in Figure 8 for different PP/PC blends. The *T*_g_ of PC decreases from 136.9 to 135.4 °C with the addition of PP, and it continues to show a decreasing trend with the addition of the three compatibilizers. The loss factor peak temperature further shifted to 134.7, 131.7, and 129.8 °C with the addition of PP-g-MA, PP-c-GMA, and E-MA-GMA for the PP/PC blends, respectively. It is well known that the *T*_g_ of PP is far lower than that of PC, and a decrease in *T*_g_ indicates an increase in the compatibility of PP/PC blends. Hence, it can be concluded from the quantitative DMA results that E-MA-GMA is the best, PP-*c*-GMA follows, and PP-*g*-MA is the worst in terms of the compatibilization effect of PP/PC blends. 

Until now, the variation of the compatibility between PP and PC can be viewed as a major reason for adjusting the foamability of the PP/PC system. The effects of the different compatibility of the PP/PC blends on the foamability are inconsistent with the mechanical properties. The improvement of compatibility is generally beneficial for the mechanical properties because it indicates an improved phase adhesion between the blend partners due to a reduction in the interfacial tension and coalescence. However, the incompatibility of the PP/PC system could provide an opportunity to improve the foamability of pure PP resin and hence lead to a finer and denser cell structure. This improvement may be attributed to the number of nucleating sites possibly increasing with the incorporation of the dispersed phase, since the interfacial boundaries between the two immiscible phases can effectively lower the critical energy barrier for bubble nucleation. If so, the foaming effect of PPC should be better than PCE. However, the opposite is actually true. Hence, there are still other possible reasons that remain undetermined, and more investigation of the forming mechanism of PCG fine foam structure needs to be carried out. A rheological test was further used to understand the foaming behavior of the PP/PC blend. 

### 3.4. Relative Rheological Behavior

**Figure 9:**
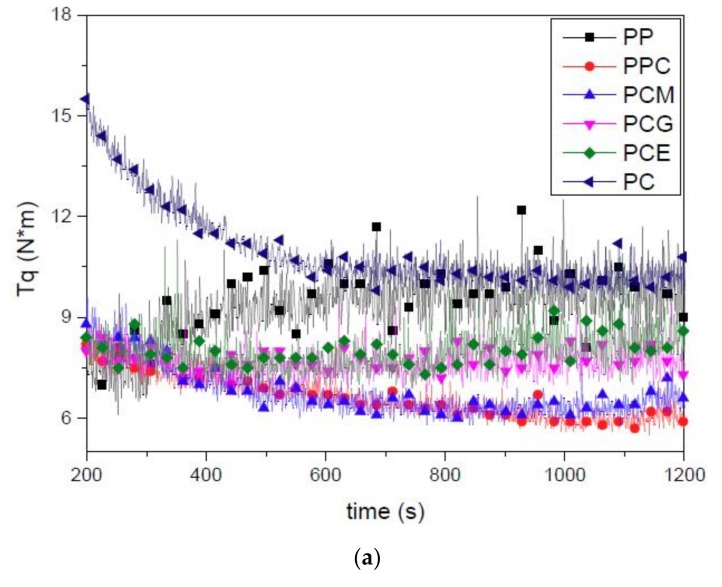

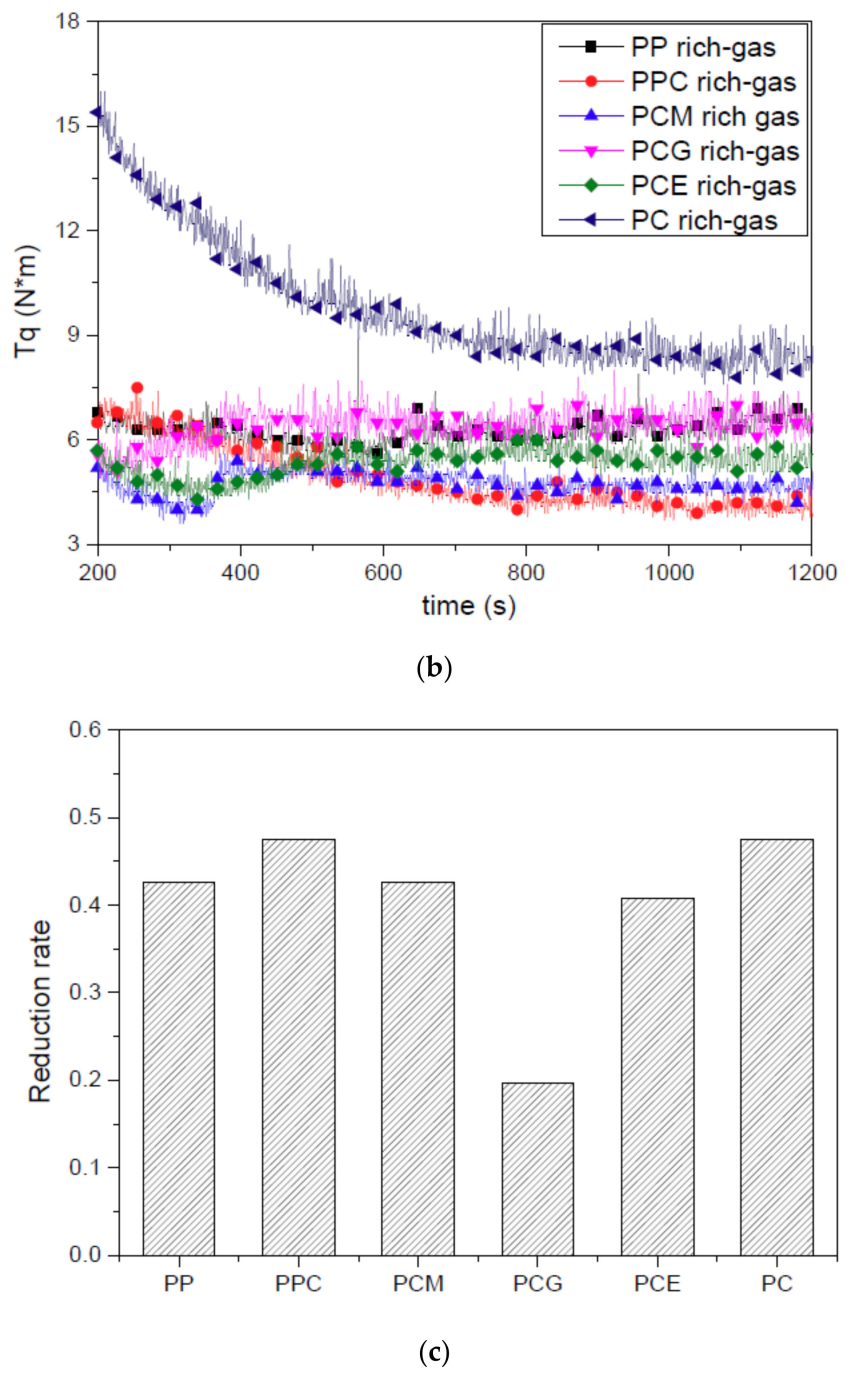
Torque rheological curves (torque vs. time) of the six no-gas PP/PC materials (**a**) and rich-gas PP/PC blends (**b**) and the reduction ratio (**c**).

It is not easy to characterize the mixture of a rich-gas melt used in foamed IM that generally involves many complex processing conditions, including not only IM processing parameters, such as variable pressure, shear rate, temperature, and temperature gradient but also the interaction between the polymer melt and the gas, such as gas release, gas dissolving in polymer melt, and injection of gas-melt solution into mold cavity. Rheological measurement is generally seen as a conventional and useful method, which has important guiding significance on the processing properties of polymers [54,55,56]. A torque rheometer, which can obtain a real-time rheological evolution of a rich-gas melt, was used to study the foaming properties of the PP/PC blends. The torque vs. time of the six PP/PC blends with and without gas is plotted in Figure 9a,b, respectively. The addition of PC, regardless of whether gas was involved, decreases the torque of pure PP resin. For the torque rheological test, the higher the balance torque is, the higher the melt viscosity. It suggested that the torque of PP decreases with the addition of PC. One can explain that it is due to the increasement of the ability of the PP chains to move freely and entangle difficultly by introducing the incompatible second phase resulting in a decrease of the PP/PC blends shear viscosity. Considering that both the PP and the PC resin have significantly higher apparent viscosity than the PP/PC blends, it can be deduced that the torque of the incompatible polymer blends did not necessarily increase or decrease linearly with blend composition. A similar conclusion had been drawn, and the effects of compatibility on the torque tested results were also reported by Wang and Li for the incompatible PP/styrene-acrylonitrile copolymer (SAN) blends [57] and Babbar and Mathur for the compatible PC/ABS blends [58]. It is also known that the viscosity difference between PP and PC is generally very large. However, the results shown in Figure 9a indicate that the viscosity of PP and PC are almost the same at the test condition. It can also be found that there is an increase in the torque when the three types of compatibilizers are present in PP/PC blends, indicating that the reaction between PP/PC can improve the interfacial tension between the blend components resulting in an increase of the PP/PC blends apparent viscosity. Furthermore, by introducing gas of the system, the ability of the polymer chains to move freely is also changed, which is reflected in a change of shear viscosity. Compared to the torque values shown in Figure 9a, the torque of the corresponding rich-gas samples shown in Figure 9b are clearly lower, which means a lower shear viscosity due to the gas dissolved in the polymer melt. 

Although a decrease in the torque with the presence of the gas is expected and can be attributed to the plasticization effect of the gas, there is still a strange phenomenon concerning the different variance of the torque between the PCG and PCE samples that needs to be clarified. To make it clearer, the difference of the torque between the rich-gas and the no-gas samples was expressed as the reduction ratio, which can be calculated by the following expression:

(3) $$R_{t}=\frac{T_{s}-T_{g}}{T_{s}}\times 100\%$$

where *R*_t_ is the reduction ratio of the torque (*T*) of rich-gas samples relative to no-gas samples, and *s* and *g* are the no-gas (solid) sample and rich-gas sample, respectively. The reduction ratio of the torque of the six PP/PC blends is plotted in Figure 9c. An interesting phenomenon can be observed from Figure 9c, whereby the addition of PP-*g*-MA or E-MA-GMA causes a clear increase in the reduction ratio of the torque and the influence of the addition of PP-*c*-GMA is relatively gentle. 

The different reduction ratio of the torque can be attributed to the different compatibility of the PP/PC blends and the presence of gas. On the one hand, for the incompatible PP/PC blend, it is more easily for the gas to assemble between the two-phase components due to the relatively low interfacial bonding between the two phases compared to the compatible system [59]. Also, the low interfacial bonding at the two-phase interface of the incompatible blends may provide a relatively easy passage for gas escape [60]. Therefore, the gas increases the chance of separation between the two phases, which leads to a higher reduction ratio. As shown in Figure 9c, the reduction ratio of PPC is obviously higher than that of pure PP resin. It is also observed from Figure 9c that the reduction ratio of PCG is smaller than that of PCM and then PPC, indicating the reduction ratio varies with the incorporation of the different compatibilizers. Compatibilized blends exhibit a higher rich-gas melt viscosity at low shear rate range used in this study with an increase in level of compatibilizing effects in the PP/PC blends. Therefore, the increase in compatibility decreases the reduction ratio of the PP/PC blends rich-gas melt viscosity. However, a strong compatible system may mean that the influence of the gas will be reflected by the blended matrix if the compatibility exceeds a limit, as occurred in the PCE samples. As a result, the reduction ratio of the PCE melt viscosity instead increases due to the excellent compatibilization as discussed before. 

It can be concluded that a moderate compatibility is beneficial for the increase of rich-gas melt viscosity. Moreover, it can also be deduced that another reason for the improved foamability of PP with the addition of PC and compatibilizer is the increase of the rich-gas melt viscosity, which results in more cell growth resistance and prevents gas escape. As discussed before, the existence of the incompatible second phase improves the foamability of pure PP resin and leads to a finer and denser cell structure. This improvement can be attributed to the number of nucleating sites possibly increasing with the incorporation of the dispersed phase, since the interfacial boundaries between the two immiscible phases can effectively lower the critical energy barrier for bubble nucleation. Furthermore, with the increase of the compatibility, although the nucleation effect of the blend possibly decreases gently in a more compatible system, the reduction of the rich-gas melt viscosity gradually decreases which facilitates the cellular structure of PP/PC blend. Therefore, the addition of PC and PP-*c*-GMA simultaneously have a comprehensive effect on the improvement of the cellular structure of PP molded samples. For the strong compatible system, PCE; however, the rich-gas melt viscosity decreases drastically resulting in its cellular structure instead deteriorating slightly. Therefore, the cellular structure of PCE is somewhat not as good as that of PCG, as discussed before. 

Therefore, according to the above analysis, the influences of PC and compatibilizer on the foaming behavior of PP can be attributed to a comprehensive effect of the interface between the PP and the PC phases and the variation of the rich-gas melt viscosity. While the foaming behavior of PP/PC blends for IM parts and the effects of compatibilization were analyzed in detail, the learning could be applied as a reference for improving the foaming behaviors of polymer blends. 

## 4. Conclusions

In this study, the foaming behavior of PP/PC and the effects of compatibilization were analyzed in detail using foaming IM processing and azodicarbonamide/zinc oxide (AC/ZnO) as blowing agents. It was found that PC can improve the cellular foam structure of a pure PP resin, and it can be attributed to a possible mechanism, i.e., the interface between the PP and PC phases increases the chance of the cell nucleation site because of the partial compatibility and the weak interaction between the two blend components. The addition of different compatibilizers makes the influence of PC on the PP foaming behavior more complex. Generally, the mechanical properties and foaming behavior of PP/PC blends improve with increasing compatibility between the PP and PC phases. However, the foaming structure cannot be further improved and can even worsen when the compatibility between the two phases exceeds a limit, which leads an increase in the reduction ratio of PP/PC melt viscosity. One can explain that it is due to a comprehensive effect of the compatibility on the rheological behavior. The compatibilizers increase the rich-gas melt viscosity of PP/PC blends and thus improves the ability of bearing the extensional forces resulting from the foaming process, which can facilitate the foaming process. Considering the PP-*c*-GMA to be the most suitable compatibilizer among the three candidates, i.e., PP-*g*-MA, PP-*c*-GMA, E-MA-GMA, which can also drastically improve the mechanical properties of PP/PC blends, the PP/PC/PP-*c*-GMA composites are preferred when foamed parts with a PP matrix are fabricated. Accordingly, an effective way of improving the foaming behavior of PP foamed injection molded components was suggested and proposed. 

## Figures and Tables

**Table 1:** The main processing parameters of the mixing and the extrusion.

Parameter	Value
Speed of mixing	480 rpm
Temperature of hopper	200~220 °C
Speed of the screw rotation	120 rpm
Speed of the take-up rolls	0.58 m/s
Temperature of take-up rolls	50 °C

**Table 2:** The selective processing parameters of injection molding.

Parameter	Value of Conventional Injection Molding	Value of Foaming Injection Molding
Melt temperature	230 °C	230 °C
Mold temperature	80 °C	80 °C
Injection pressure	90 MPa	90 MPa
Injection rate	50 cm^3^/s	50 cm^3^/s
Packing pressure	75 MPa	10 MPa
Packing time	5.0 s	1.0 s
Cycle time	40 s	40 s

**Table 3:** Density of the six kinds of PP/PC solid and foamed molded samples.

Samples	Solid (Unit: kg/m^3^)	Foamed (Unit: kg/m^3^)
PP	908	819
PPC	1135	1011
PCM	1131	1006
PCG	1130	1004
PCE	1131	1007
PC	1211	1086
